# From the Editor's desk

**DOI:** 10.4103/0974-1208.44110

**Published:** 2008

**Authors:** Kamini A. Rao

**Affiliations:** Editor-in-Chief, Journal of Human Reproductive Sciences. E-mail: kambacc@vsnl.com

It is my proud privilege to be writing this editorial for the second issue of Journal of Human Reproductive Sciences. It has always been my intention to provide the readers of JHRS with a varied selection of relevant and interesting articles in each issue of the general. At the outset, I must first stress that I consider all papers that reach the publication stage of this journal to be of high quality and importance. Our reviewing process is quite stringent and is a testimonial to this. The papers selected are those that we have found to be particularly interesting and we hope that perhaps you will too.

The two review articles on Endometriosis by Ian Fraser of Australia and Ovarian Cryopreservation by Dr. Balaji of Singapore are both interesting and informative. Our sincere thanks to Judy Passlow, publisher of “Medicine Today” for granting us permission to reproduce the article on Endometriosis by Ian Fraser. Over the last few years, fertility preservation in cancer patients by cryostorage of ovarian tissue has become a part of clinical practice. There is however a risk that cancer cells are present in the stored tissue and that the cancer could recur after transplantation. To increase the safety of the procedure, methods to detect disease in the ovaries and malignant cells in stored tissue are indicated.

Outcome in Impaired Spermatogenesis, Polycystic Ovarian Disease and Change in Serum Calcium, Manganese and Phosphorous levels in different phases of the menstrual cycle, form the lead articles for this issue along with four case reports. I do hope you will enjoy reading this issue as much as we enjoyed bringing it to you.

